# Will nanomedicine become a good solution for the cardiotoxicity of chemotherapy drugs?

**DOI:** 10.3389/fphar.2023.1143361

**Published:** 2023-05-04

**Authors:** Yichuan Jiang, Yueyao Jiang, Min Li, Qian Yu

**Affiliations:** ^1^ Department of Pharmacy, China-Japan Union Hospital, Jilin University, Changchun, China; ^2^ Pharmacological Experiment Center, School of Pharmaceutical Sciences, Jilin University, Changchun, China

**Keywords:** nanomedicine, chemothearpy, cardio-oncology, paclitaxel, doxorubicin, cardiotoxcity

## Abstract

Cancer is one of the leading causes of death worldwide, and with the continuous development of life sciences and pharmaceutical technology, more and more antitumor drugs are being used in clinics to benefit cancer patients. However, the incidence of chemotherapy-induced cardiotoxicity has been continuously increasing, threatening patients’ long-term survival. Cardio-oncology has become a research hot spot, and the combination of nanotechnology and biomedicine has brought about an unprecedented technological revolution. Nanomaterials have the potential to maximize the efficacy and reduce the side effects of chemotherapeutic drugs when used as their carriers, and several nano-formulations of frequently used chemotherapeutic drugs have already been approved for marketing. In this review, we summarize chemotherapeutic drugs that are highly associated with cardiotoxicity and evaluate the role of nano-delivery systems in reducing cardiotoxicity based on studies of their marketed or R&D nano-formulations. Some of the marketed chemotherapy drugs are combined with nano-delivery systems that can effectively deliver chemotherapy drugs to tumors and cannot easily penetrate the endothelial barrier of the heart, thus decreasing their distribution in the heart and reducing the cardiotoxicity to some extent. However, many chemotherapy nanomedicines that are marketed or in R&D have not received enough attention in determining their cardiotoxicity. In general, nanomedicine is an effective method to reduce the cardiotoxicity of traditional chemotherapy drugs. However, cardiovascular complications in cancer treatment are very complex diseases, requiring the application of multiple measures to achieve effective management and prevention.

## 1 Introduction

With the continuous improvement of tumor diagnosis and treatment in recent years, the survival period of tumor patients has been extended, and the life expectancy of cancer patients has increased steadily. Taking breast cancer as an example, as of 2018, the five-year survival rate of breast cancer has reached more than 85% in most Western European and North American countries, and more than 80% in many developing countries such as China and Turkey (Zaidi and Dib, 2019). However, the number of new cancer cases is rising worldwide, forming a large and growing group of cancer survivors. In 2016, there were 1.55 million cancer survivors in the United States, which is expected to exceed 2.61 million by 2040 (Bluethmann et al., 2016). More and more cancer survivors are troubled by long-term complications related to cancer treatment, including cardiovascular disease (CVD), sub-/infertility, and neurotoxicity. Among them, CVD is gradually becoming one of the main causes of death for cancer survivors. One study of about 3.23 million cancer patients in the United States from 1973 to 2012 showed that 38.0% died of cancer and 11.3% died of CVD (Sturgeon et al., 2019). Therefore, cardio-oncology has become a hot emerging interdisciplinary subject.

In the 1970s, there were many reports of heart failure in patients after anthracycline chemotherapy (Chlebowski, 1979), which was the first chemotherapy drug to be assessed for cardiotoxicity. In actuality, each chemotherapeutic agent has unique cardiac effects as well as the ability to potentiate the adverse effects of other agents. Radiation therapy also plays an important role in magnifying toxicity. The therapeutic options for patients with cancer now include increasingly complex combinations of medications, radiation therapy, and surgical intervention. Many of these treatments have important potential adverse cardiac effects and are likely to have significant effects on patient outcomes (Zhu et al., 2021). At the beginning of the 21st century, the University of Texas M.D. Anderson Cancer Center established the Department of Cardiology, marking the rise of cardio-oncology, whose mission is to study the diagnosis, pathogenesis, and management of cardiovascular complications in cancer therapy.

Today, the use of traditional chemotherapy drugs, especially anthracycline chemotherapy drugs, is still the most important cause of tumor-related CVDs (Lenneman and Sawyer, 2016). Although new targeted antitumor drugs continue to make breakthroughs, chemotherapy drugs are still the cornerstone of the medical treatment of cancer. The cardiotoxicity of chemotherapy drugs can cause patients to die directly from adverse cardiovascular events, or the treatment of the tumor can be interrupted or terminated due to CVD progression. Therefore, reducing the cardiotoxicity of chemotherapy drugs is an important direction of tumor drug research and development.

**FIGURE 1:**
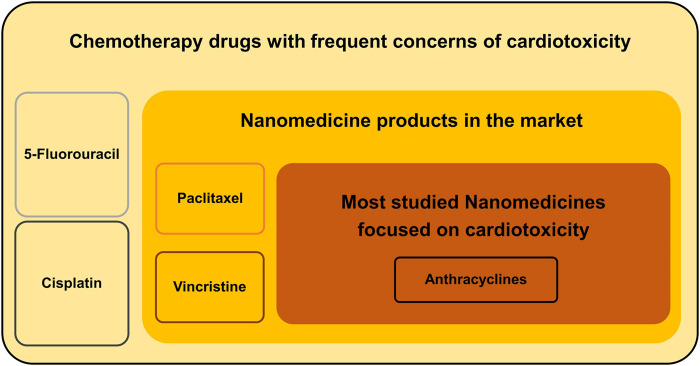
Three levels of applications of nanotechnology to reduce the cardiotoxicity of chemotherapy drugs.

At the beginning of the 21st century, the integration of nanotechnology and biotechnology greatly promoted the progress of medicine. In recent years, nanomedicines have been applied to the treatment of various diseases, especially cancer, which is mainly attributed to their biological advantages, such as improved solubility and pharmacokinetics profile, enhanced bioavailability, increased selectivity, and reduced toxicity (Shi et al., 2017). Since the FDA approved the first nanomedicine in 1995 (Barenholz, 2012), several of them have been approved and marketed for cancer treatment. Considering the worldwide trend of the aging population, the number of cancer patients with cardiovascular-related adverse events will continue to increase. Whether nanotechnology can improve antitumor efficacy and cardiovascular system protection is an important research direction, prompting an evaluation and systematic review of the current status of nanotechnology applications in the prevention and treatment of chemotherapy-induced cardiotoxicity. Therefore, this review focuses on studies of nanomedicine-based chemotherapy drugs either in the market or in R&D and summarizes whether the application of nanotechnology can reduce their cardiotoxicity. There are three levels of these nanomedicines, as shown in Figure 1: 1) those without nanomedicines based on them in the market, such as 5-FU and cisplatin; 2) those with nanomedicines based on them in the market but with no clinical trials focused on cardiotoxicity, such as paclitaxel and vincristine; and 3) those with nanomedicines based on them in the market that have also been proved to reduce cardiotoxicity by clinical trials, such as anthracyclines.

## 2 Antitumor antibiotics

### 2.1 Cardiotoxicity of antitumor antibiotics

Antitumor antibiotics include anthracyclines and polypeptide antibiotics. Cardiotoxicity is not one of the major side effects of polypeptide antibiotics such as bleomycin and actinomycin D. Anthracyclines such as doxorubicin, daunorubicin, epirubicin, and idarubicin are known to be very effective and widely used in chemotherapy. The combination of anthracyclines with other chemotherapeutic agents, targeted agents, and radiotherapy has been the preferred first-line treatment option for various solid tumors and hematological malignancies (Alavi and Varma, 2020). However, the application of anthracycline is often complicated by dose-dependent cardiomyopathy and congestive heart failure (Vejpongsa and Yeh, 2014). The exact mechanism of anthracycline-induced cardiotoxicity remains uncertain, but the most widely accepted hypothesis is that the quinone moiety that is common to all anthracyclines acts as a free radical that reacts with oxygen to form the superoxide anion, which is disproportionated to hydrogen peroxide, leading to an increase in reactive oxygen species (ROS). Oxidative stress can also occur through the induction of nitric oxide synthase, leading to the production of reactive nitrogen, particularly peroxynitrite. These reactive oxygen and reactive nitrogen species can lead to mitochondrial functional impairment, energy imbalance, and even cardiomyocyte apoptosis (Henriksen, 2018). In addition, many recent studies have identified topoisomerase II β (Top II β) as a possible key mediator of anthracycline-induced cardiotoxicity, which acts by altering the topology of DNA during its replication, chromosome condensation, and sister chromatid duplex separation. There are two topoisomerase isoforms in mammals: II α and II β. Topoisomerase II α, found primarily in proliferating cells, is required for DNA replication and thought to be the target of anthracyclines in their antitumor effects. In contrast, Top II β is present in all quiescent cells, including cardiomyocytes. Anthracyclines cause DNA double-bond breakage by inhibiting Top II β, leading to cardiomyocyte death (Zhang et al., 2012; Uddin et al., 2022).

The main advantages of nano-delivery systems are effective targeting of the therapeutic site, delayed drug release, and extended drug half-life. Based on these advantages, it is feasible to employ nano-delivery systems for anthracyclines to reduce their dose-dependent cardiotoxicity (Vargason et al., 2021). So far, a number of nanotechnology-based anthracycline products have been marketed, and several advantages have been reported, such as efficient drug loading and encapsulation, targeted/controlled drug release, and reduced cardiotoxicity. As nanotechnology continues to evolve, other, more advanced nano-delivery systems designed for anthracyclines are currently entering the R&D stage. In the following section, we review the role of nanotechnology in improving the cardiotoxicity of anthracyclines based on marketed and experimental formulations.

### 2.2 Nanotechnology-based marketed anthracycline products

#### 2.2.1 Doxil/Lipo-Dox

Doxorubicin is the most widely used anthracycline drug with significant therapeutic efficacy in a variety of cancers, including leukemia, lymphoma, and solid tumors such as liver, ovarian, breast, thyroid, and lung cancers. However, its clinical use has been limited by dose-dependent, long-term, and potentially lethal cardiotoxicity. Doxil^®^, also known as Caelyx, was the first FDA-approved nanomedicine for cancer therapy (Barenholz, 2012). It is PEGylated liposomal doxorubicin, clinically indicated for treating acquired immune deficiency syndrome (AIDS)-related Kaposi’s sarcoma, metastatic breast cancer, advanced ovarian cancer, and multiple myeloma (Duggan and Keating, 2011). The liposome consists of a phospholipid, hydrogenated soy phosphatidylcholine, cholesterol, and N-(carbonyl-methoxy polyethylene glycol 2000)-1,2-distearoyl-sn-glycero-3-phosphoethanolamine sodium salt (MPEG-DSPE), and doxorubicin is loaded in the hydrophilic core of the liposome.

Lipo-Dox is a next-generation PEGylated liposomal doxorubicin similar to Doxil produced by Sun Pharma, which was allowed temporary importation by the FDA in response to critical shortages of Doxil. PEG-distearoyl phosphatidylethanolamine is used as the hydrophilic outer layer, identical to Doxil, and differs only in the composition of the lipid membrane, which in Lipo-Dox is composed of distearoyl phosphatidylcholine (DSPC) (Berger et al., 2014). Compared to Doxil, Lipo-Dox shows a lower clearance, longer half-life, and smaller volume of distribution, indicating its higher stability in plasma. However, there is a higher incidence of severe stomatitis in patients treated with Lipo-Dox (Hong and Tseng, 2001).

The nanometer size of the liposome and the reticuloendothelial system (RES) shielding properties attributed to PEGylation allow Doxil and Lipo-Dox to have increased circulation time and enhanced drug accumulation at the tumor site. PEGylated liposomal formulation is one of the first classes of nano-delivery systems that have successfully translated into clinical applications. It consists of one or several lipid bilayers, ranging in size from 20 nm to 1,000 nm, where hydrophilic drugs can be enclosed in the aqueous inner region of the liposome, and hydrophobic drugs can be wrapped in the hydrocarbon chain region of the lipid bilayer. Doxil and Lipo-Dox, both PEGylated liposomal formulations of doxorubicin (PLD), exploit the enhanced permeation and retention effect (EPR) to reach tumor tissue by passive targeting of cancer cells, and are more effective than conventional doxorubicin. A study was designed to assess whether PLD has a superior cardiac safety profile as compared with doxorubicin in the first-line treatment of women with metastatic breast cancer (MBC). Women with MBC and normal cardiac function (*n* = 509) were randomized to receive PLD 50 mg/m^2^ (every 4 weeks) or doxorubicin 60 mg/m^2^ (every 3 weeks), and cardiotoxicity was assessed based on the reduction in left ventricular ejection fraction in relation to cumulative anthracycline dose. The results showed that the total risk of cardiotoxicity was significantly higher in the doxorubicin-treated patients than in the PLD-treated ones (HR = 3.16; 95% CI 1.58–6.31; *p* < 0.001). Doxorubicin treatment was more frequently associated with alopecia (overall, 66% versus 20%; significantly, 54% versus 7%, respectively), nausea (53% versus 37%), vomiting (31% versus 19%), and neutropenia (10% versus 4%) than PLD (O’Brien et al., 2004). A retrospective cohort study of 56 patients receiving large cumulative doses of PLD (450–1,400 mg/m^2^) demonstrated a lower risk of heart failure induced by PLD compared to doxorubicin (Skubitz et al., 2017). Savani et al. (2021) reported a case of a 50-year-old man with terminal retroperitoneal liposarcoma who did not have detectable cardiotoxic events after 84 months of PLD treatment. Another study monitored 14 patients with recurrent gynecologic cancers who received long-term treatment with large cumulative doses of PLD. The mean PLD treatment duration was 23.6 ± 10.8 months (range: 13–57 months), and the cumulative dose was 1,387 ± 483 mg (range: 780–2,538 mg). The results showed that despite long-term treatment with large cumulative doses of PLD, no prevalent or incidental cardiotoxic events were observed (Blank et al., 2017). Ansari et al. (2017) reviewed clinical trials of PLD monotherapy in women with metastatic breast cancer and found a substantially lower incidence of cardiotoxicity in patients treated with PLD compared to conventional doxorubicin. Another study investigated the therapeutic effect of a DRCOP (doxorubicin + cyclophosphamide + vincristine + dexamethasone) regimen based on PLD compared with conventional doxorubicin on 80 patients with diffuse large B cell lymphoma. A significant reduction in cardiotoxicity (e.g., myocardial infarction, myocardial injury, and abnormal electrocardiogram) was detected in the PLD group compared with the conventional doxorubicin group. The DRCOP scheme based on PLD can obtain a better therapeutic effect, with higher effective and survival rates and lower cardiotoxicity (Liu and Zhang, 2021).

#### 2.2.2 Myocet^®^

Myocet^®^ is a non-PEGylated liposome of doxorubicin (NPLD) developed by the Liposome Company, Inc. (now Elan Pharmaceuticals), which was commercially approved in 2000 as a first-line therapy in combination with cyclophosphamide for metastatic breast cancer in Europe and Canada. The pharmacokinetics of Myocet^®^ are different from conventional doxorubicin. Plasma levels of total doxorubicin are substantially higher in Myocet^®^ treatment than conventional doxorubicin, whereas the peak plasma levels of free doxorubicin are lower. After Myocet^®^ treatment, 85%–93% of the doxorubicin in the plasma was encapsulated, resulting in a different tissue distribution pattern for doxorubicin. Dogs receiving Myocet^®^ showed doxorubicin levels in myocardial tissues that were approximately 67% of those in conventional doxorubicin treatment (Swenson et al., 2001), suggesting that Myocet^®^ can potentially produce less myocardial toxicity than conventional doxorubicin.

In a phase III trial (Batist et al., 2001), patients received cyclophosphamide 600 mg/m^2^ plus NPLD 60 mg/m^2^ (*n* = 142) or conventional doxorubicin 60 mg/m^2^ (*n* = 155) for a median of 6 cycles/patient. Antitumor efficacy was comparable between the two groups, with an objective response rate of 43% in both groups, a median time to progression of 5.1 versus 5.5 months, a median time to treatment failure of 4.6 versus 4.4 months, and a median survival time of 19 versus 16 months, respectively. Significantly fewer patients developed cardiotoxicity in the NPLD group (6% versus 21%; *p* = 0.0002). There were five cases of congestive heart failure (CHF) in the conventional doxorubicin group and none in the NPLD group (*p* = 0.02). The estimated median cumulative lifetime dose of doxorubicin at the first occurrence of cardiotoxicity (defined as significant left ventricular ejection fractions changes or CHF) was >2,220 mg/m^2^ in the NPLD group versus 480 mg/m^2^ in the conventional doxorubicin group. Another study (Harris et al., 2002) compared NPLD (75 mg/m^2^; *n* = 108) with conventional doxorubicin (75 mg/m^2^; *n* = 116) as a monotherapy for a median of four cycles. The overall response rate was 26% in both groups, and time to disease progression was 3.8 months in the NPLD arm versus 4.3 months in the doxorubicin arm. Cardiac events sufficient for the removal of a patient from the trial were twice as likely in the doxorubicin group (13% versus 29%; *p* = 0.0001). There were nine cases of CHF in the conventional doxorubicin group and two in the NPLD group, and the estimated median cumulative lifetime dose of doxorubicin at the first occurrence of cardiotoxicity was 785 mg/m^2^ versus 533 mg/m^2^ (log rank *p* = 0.0001), respectively.

The impact of using NPLD in patients with high-risk factors (previous anthracycline exposure or history of cardiac disease) was evaluated in a subset analysis of the data obtained from two Phase III studies (Batist et al., 2006). This subset analysis was performed on pooled data including 68 patients who had received previous adjuvant conventional doxorubicin. Even though patients in the NPLD group had received a greater cumulative dose of conventional anthracycline compared with conventional doxorubicin (308 mg/m^2^ versus 225 mg/m^2^, respectively), the incidence of cardiac events was significantly lower in the former (22% versus 39%, *p* = 0.01). Furthermore, the median lifetime dose at the onset of cardiotoxicity was 780 mg/m^2^ for NPLD versus 580 mg/m^2^ for conventional doxorubicin. Therefore, evidence shows that NPLD has significant advantages over conventional doxorubicin in terms of similar or better oncological efficacy with a significantly lower incidence of cardiotoxicity, even in patients with high-risk factors from previous anthracycline therapy.

#### 2.2.3 DaunoXome

Daunorubicin is one of the most well-known anthracyclines and an important drug in many treatment protocols in both adult and pediatric oncology. Unfortunately, as for all anthracyclines, cumulative high dose is the main risk factor for (irreversible) congestive heart failure. In an attempt to improve the pharmacokinetics and chemical stability of free daunorubicin, as well as to reduce toxicity, DaunoXome, a liposomal formulation of daunorubicin, was created and developed by NeXstar Pharmaceuticals, USA and sold by Gilead Sciences (now owned by Galen Pharmaceuticals, United States). DaunoXome was approved for marketing in 1996 for the therapy of Kaposi sarcoma-associated with human immunodeficiency virus and acute myeloid leukemia. The drug consists of the citrate salt of daunorubicin encapsulated within highly stable lipid vesicles (liposomes) comprising a bilayer membrane of distearoyl phosphatidylcholine and cholesterol. These remarkably stable vesicles prevent the rapid and extensive uptake of the entrapped drug by the reticuloendothelial system and its diffusion in most cells. Due to the slow distribution of liposomes, DaunoXome demonstrates a lower clearance compared with free daunorubicin (Hempel et al., 2003; Pea et al., 2003). While in circulation, DaunoXome helps to protect the entrapped daunorubicin from chemical and enzymatic degradation, minimizes protein binding, and generally decreases uptake by normal (non-reticuloendothelial system) tissues, such myocardial cells (Pouna et al., 1996; Löfgren et al., 2007). A phase I study of liposomal daunorubicin in patients (n = 14) older than 60 years with relapsed and refractory acute myeloid leukemia was conducted to investigate the toxicity of DaunoXome. The mean cumulative dose of DaunoXome administered was 340 mg (range: 120–1,200 mg). DaunoXome appeared to be less cardiotoxic compared with free daunorubicin, even at the highest cumulative doses (1,200 mg/m^2^) (Bieker et al., 2003). Another study evaluated the safety, pharmacokinetics, and efficacy of DaunoXome in patients with AIDS-related Kaposi’s sarcoma; even at cumulative doses of DaunoXome greater than 1,000 mg/m^2^, no significant declines in cardiac function were observed (Gill et al., 2016). In summary, DaunoXome, compared with free daunorubicin, seems to be characterized by higher tumor cell delivery, improved pharmacokinetics, a better therapeutic index, and less cardiotoxicity.

### 2.3 Nanotechnology-based formulations of anthracyclines in R&D

#### 2.3.1 Doxorubicin

With the combination of genetic engineering and nanotechnology, immunoliposomes (considered third-generation liposomes) have been developed for the treatment of cancer, in which monoclonal antibodies or their fragments are conjugated to the surface of liposomes. As a result, immunoliposomes themselves have complement-dependent cytotoxicity and/or antibody-dependent cell-mediated cytotoxicity and can also produce synergistic cytocidal effects with encapsulated chemotherapy drugs. In addition, due to the better targeting of immunoliposomes, the toxic effects of chemotherapy drugs on non-tumor sites can be reduced (Kirpotin et al., 2006; de Freitas et al., 2023). Reynolds et al. developed HER2-targeted liposomal doxorubicin and estimated the doxorubicin exposure in human stem cell–derived cardiomyocytes. They demonstrated that HER2-targeted liposomal doxorubicin was rarely uptaken into cardiomyocytes, resulting in little or no evidence of cardiomyocyte cell death or dysfunction. In a mouse model, the lower accumulation of doxorubicin in the myocardium was treated with HER2-targeted liposomal doxorubicin compared with free doxorubicin (Reynolds et al., 2012).

Polymeric micelles are effective delivery systems for hydrophobic anticancer drugs and exhibit prolonged circulation time in the blood and enhanced permeability in tumor tissues. Different approaches, including non-covalent (hydrophobic and π–π interactions) and chemical (covalent binding of the drug to the polymer backbone and/or crosslinking of the core/shell) strategies have been used to improve stability in circulation and to retain the loaded drug in the PM (Zhang et al., 2014; Varela-Moreira et al., 2017). A micellar drug delivery system was synthesized by conjugating hydrophobicdoxorubicin and β-sitosterol to hydrophilic N-(2-hydroxypropyl) methacrylamide polymer backbone via pH-sensitive hydrazone linkages, which provided significantly higher tumor accumulation and antitumor rate in a xenograft hepatocarcinoma-bearing mice model, and exhibited no necrosis, hyperemia, or inflammation in heart tissue (Zhou et al., 2014).

The key characteristics of dendrimers that make them potential drug carriers include their excellent uptake by cells, high density, multiple functionalities, and their ability to carry higher quantities of drugs with relatively higher molecular weights. The ability of modified dendrimers to target tumor tissue is greatly enhanced, whereas the distribution of drugs in healthy myocardium is reduced. Poly (2-methacryloyloxyethyl phosphorylcholine)–modified third-generation poly (amidoamine) dendrimers (PAMAM-PMPC) (Ban et al., 2021) loaded with doxorubicin alleviated weight loss in tumor-inoculated mice and reduced the cardiotoxicity of doxorubicin. Upon a histological examination of heart sections, the free doxorubicin group had obvious necrotic cells and myocardial damage, whereas the doxorubicin-loaded PAMAM-PMPC group displayed no abnormalities, necrosis, or inflammation in the heart. Loading doxorubicin onto fifth-generation PAMAM dendrimers targeting hepatic cancer cells via N-acetylgalactosamine ligands showed controllable doxorubicin release and toxicity towards hepatic cancer cells comparable to free doxorubicin. Doxorubicin dendrimers incubated with human-induced pluripotent stem cell–derived cardiomyocytes did not reduce monolayer viability, and induced apoptosis, contrary to the effects of free doxorubicin. Additionally, doxorubicin dendrimer–treated mice maintained cardiac function following intraperitoneal injection of 1 mg/kg doxorubicin for 21 days (Kuruvilla et al., 2017).

Hydrogels have excellent biocompatibility, negligible cytotoxic biodegradability, prominent drug-encapsulating capability, and controlled release. Hydrogels also allow for local injection owing to their preferable permeability into tumor tissues and sensitivity to environmental stimuli. Silver nanoparticle (NP)-embedded hydrogels and colloidal mesoporous silica NP-embedded Pluronic F127 hydrogels were reportedly combined with doxorubicin; the doxorubicin NP-embedded hydrogels reduced malondialdehyde (MDA) and total nitrate/nitrite levels and boosted superoxide dismutase (SOD) and glutathione (GSH) levels in rat cardiac tissues, decreased inflammatory infiltration, and improved the heart function (Silveira et al., 2016; Mansour et al., 2018). Recently, other nanoformulations were employed to deliver doxorubicin. A study on the cardiotoxicity of bicontinuous cubic liquid crystalline NPs carrying doxorubicin (DOX-LCNPs) was explored in a dimethyl phenylanthracene (DMBA)-induced breast cancer model, where lower levels of CK-MB, LDH, and MDA, and higher levels of GSH and SOD were found in the DOX-LCNP treatment group compared with doxorubicin treatment. Histopathological examination showed that doxorubicin-induced excessive vacuoles and the destruction of severe fine structure were rarely observed in the DOX-LCNP treatment group, indicating that the incorporation of doxorubicin in the novel LCNPs improved doxorubicin-induced cardiotoxicity (Swarnakar et al., 2014).

#### 2.3.2 Pirarubicin

Pirarubicin is a semi-synthetic anthracycline of doxorubicin that is reported to have much more rapid intracellular uptake, more effective antitumor activity, and lesser cardiac toxicity compared to doxorubicin. However, long-term use of pirarubicin causes bone marrow suppression and severe cardiotoxicity. Anees et al. (2022) developed pirarubicin encapsulated with biodegradable polymeric NPs to reduce off-target toxicity and enhance antitumor efficacy, which exhibited higher antitumor efficacy than free drugs in Ehrlich Ascites Carcinoma–bearing BALB/c mice. Treatment with free pirarubicin caused severe cardiotoxicity indicated by atrophy and hemorrhage in the myocardium, whereas no adverse effects on cardiac muscles was seen in mice treated with pirarubicin-encapsulated NPs.

#### 2.3.3 Epirubicin

Yu et al. (2018) prepared and evaluated EPI/siBCL-2 dual-loaded lipid-NPs (MEND) formed by using an acid-cleavable ketal-containing poly to bind siBCL-2, and the KPAE/siBCL-2 complexes were further coated by an epirubicin (EPI)–containing lipid. Co-delivering epirubicin and siBCL-2 simultaneously via lipid-NPs showed enhanced inhibition efficiency toward HepG2 cells. A living-animal imaging system observed that PI/Cy5-siRNA-MEND was mainly distributed in the liver and also a small part of the kidney, but there was no distribution in the heart of BALB/c nude mice, which indicated no off-targeted toxicity toward the heart. Another study by Takahashi et al. (2013) demonstrated that epirubicin-incorporated micelles (NC-6300) could decrease cardiotoxicity and strengthen antitumor effects. Echocardiography in C57BL/6 mice treated with epirubicin exhibited visibly decreased fractional shortening and diminished left ventricular ejection fraction. In contrast, the cardiac functions of the NC-6300-treated mice were similar to those of the normal control mice.

## 3 Antitumor drugs extracted from plants

### 3.1 Cardiotoxicity of antitumor drugs extracted from plants

Antitumor drugs extracted from plants mainly include taxanes, vinblastines, camptothecins, and podophyllotoxins, with taxanes and vinblastines having obvious cardiotoxicity.

Paclitaxel was originally identified from the bark and needles of plants in the *Taxus* genus. It inhibits tumor growth mainly by promoting microtubule protein polymerization and keeping microtubule proteins stable, thereby inhibiting cell mitosis. Currently, paclitaxel is widely used in the clinical treatment of various solid tumors such as ovarian, cervical, esophageal, breast, and gastric cancers. Due to its low water solubility, paclitaxel is formulated in a mixture of Cremophor EL and dehydrated ethanol (50:50, v/v)—a combination known as Taxol. However, Cremophor EL is not biologically inert and leads to several adverse biological effects (Gelderblom et al., 2001). Clinically, Taxol often causes asymptomatic reversible bradycardia, blood pressure changes, arrhythmia, myocarditis, and pericarditis (Gallego-Jara et al., 2020), among which arrhythmia is the most common adverse cardiac reaction. The main clinical manifestation is asymptomatic bradycardia, and most of it occurs during medication (Guglin et al., 2009). Combining paclitaxel and anthracyclines has been shown to increase the risk of heart failure (Perez, 1998; 2001; Danesi et al., 1999; Agunbiade et al., 2019). Although the cardiac toxicity caused by paclitaxel is reversible, its specific mechanism is not clear; it may include damage to cardiac cells and endothelial function, and also the interference of cardiac electrical conduction function, leading to arrhythmia. The mechanism of the increased cardiac toxicity of the combination of Taxol and anthracyclines is that paclitaxel prolongs the metabolic time of anthracyclines and their metabolites *in vivo* (Jain and Aronow, 2019).

Vincristine is an antitumor vinca alkaloid that inhibits microtubule formation in the mitotic spindle, resulting in an arrest of cell division at the metaphase stage. Vincristine is mainly used for the treatment of acute lymphoblastic leukemia, but it is also effective in other acute leukemias, Hodgkin’s disease, lymphosarcoma, reticulocyte sarcoma, and breast cancer. Vincristine is a cell cycle–specific drug and is often required in combination antitumor regimens with cell cycle non-specific drugs; therefore, most of the clinical data collected on the cardiotoxicity of vincristine are on its co-action with other chemotherapeutic agents (Lichtman et al., 2000). Damage to blood vessels (e.g., phlebitis) is one of the most important side effects of vinblastine (de Lemos, 2005).

### 3.2 Nanotechnology-based marketed products of antitumor drugs extracted from plants

#### 3.2.1 Abraxane

Abraxane is an albumin-bound, solvent-free paclitaxel NP manufactured by Abraxis BioScience that first received FDA approval in 2005 for breast cancer therapy, and then, in 2012, was approved in combination with carboplatin as a first-line therapy for advanced non-small-cell lung cancer (NSCLC). In 2013, an indication of Abraxane was expanded by the FDA to include advanced pancreatic cancer. Albumin, as a transporter of many molecules such as hormones, fatty acids, bilirubin, calcium, and zinc in the blood, has many binding sites for hydrophobic molecules (both specific and non-specific). This property—in addition to its tendency to accumulate in tumors either due to the EPR effect or by caveolae-mediated transport through the endothelium—makes albumin an effective agent for the delivery of hydrophobic molecules to tumors. Based on this natural affinity, nab technology exploits non-covalent hydrophobic interactions to form nab-paclitaxel. Due to the solvent-free composition of Abraxane, it overcomes the toxicities associated with Cremophor EL. Moreover, albumin present in the formulation can initiate transcytosis mediated by albumin receptor gp60 through endothelial cells. Furthermore, the ability of albumin to bind with the secreted proteins overexpressed in many tumors results in a higher accumulation of the drug in tumor cells, which also increase its efficacy. In a phase III randomized trial, during a 30-min infusion of Abraxane, hypotension occurred in 5% of patients with metastatic breast cancer, and bradycardia occurred in <1% of patients. These changes in vital signs most often caused no symptoms and required neither specific therapy nor treatment discontinuation. During post-marketing surveillance, rare reports of congestive heart failure and left ventricular dysfunction have been observed among individuals receiving Abraxane (Guarneri et al., 2012). Abraxane has a significantly larger maximum tolerated dosage compared with Taxol, and it mitigates numerous Taxol-associated adverse effects such as hypersensitivity reactions, neutropenia, hypotension and bradycardia (Ibrahim et al., 2002; Gradishar et al., 2005).

#### 3.2.2 Genexol-PM

Genexol-PM is a lyophilized polymeric micellar intravenous injection containing paclitaxel developed by Samyang Corporation. It was launched in 2007 in the Korean market to treat breast cancer and NSCLC. Similar to Abraxane, this formulation was also developed as a Cremophor EL–free micellar preparation to eliminate its adverse toxic effects. The hydrophilic shell of Genexol-PM prevents the absorption of RES and increases its circulation time. With a particle size of 20–50 nm, Genexol-PM exhibits enhanced solubility and efficacy with reduced toxicity. Due to its lower toxicity, Genexol-PM has a maximum tolerated dose that is about 2-3 times higher than Taxol. It also shows higher accumulation in tumor tissue with excellent drug efficacy. In a phase III trial comparing Genexol-PM and Taxol as first-line treatments for advanced NSCLC, Genexol-PM plus cisplatin yielded superior objective response rate (50% versus 26%; rate ratio: 1.91; *p* < 0.0001) and progression-free survival (6.4 months versus 5.3 months; hazard ratio: 0.63; *p* = 0.0001) results compared with the Taxol plus cisplatin group. The incidence of treatment-related serious adverse events (9% versus 18%; *p* = 0.0090) was significantly lower in the Genexol-PM plus cisplatin group than in the Taxol plus cisplatin group, and no clear cardiotoxicity-related adverse events were observed (Shi et al., 2021). It is worth noting that polymeric colloidal paclitaxel was found to be associated with a high accumulation of paclitaxel in the heart; therefore, the cardiotoxicity risk of these agents may deserve further investigation (Li et al., 2018).

#### 3.3.3 Marqibo

Marqibo, also called Optisomes, is a liposome-based formulation of vincristine sulfate developed by Talon Therapeutics and approved by the FDA in 2012 for the treatment of Philadelphia chromosome-negative acute lymphoblastic leukemia (ALL) in adult patients suffering from two or more relapses or in which the disease has progressed after two or more antileukemia treatments. It comprises cholesterol and sphingomyelin at a molar ratio of 40:60 and exhibits an acceptable particle size (<100 nm). The composition of the vesicles and the small particle size both reduce protein binding and prolong the circulation time in the blood. Marqibo showed higher area under the curve of drug concentration versus time (AUC) as well as lower clearance as compared to conventional vincristine, with about 95% drug loading and slow drug release into tumor vasculature (Silverman and Deitcher, 2013). Marqibo exhibited a similar toxicity profile at approximately twice the dose of the pure drug in a phase II clinical trial in patients with refractory aggressive non-Hodgkin lymphoma (Rodriguez et al., 2009; Hagemeister et al., 2013). However, no direct studies on the cardiotoxicity of Marqibo have been reported.

### 3.3 Nanotechnology-based formulations of antitumor drugs extracted from plants in R&D

In addition to the already marketed paclitaxel nanoformulations described above, a recent study introduced a novel liposomal paclitaxel (lipo-PTX) composed of paclitaxel/lipid at a molar ratio of 2:98. The lipo-PTX formulation possessed a similar therapeutic efficacy to Taxol in various cancer models. The cardiovascular impacts were elucidated in mice treated with Taxol, which exhibited abnormal ECG recordings, including an irregular heartbeat and apparent QTc prolongation, indicating cardiac arrhythmia. Conversely, there were no obvious aberrations in ECG recordings following lipo-PTX treatment (Huang et al., 2018).

## 4 Other chemotherapy drugs

### 4.1 Other chemotherapy drugs with potential cardiotoxicity

5-fluorouracil (5-FU), an antimetabolic drug, is commonly used in many solid tumors such as digestive tract tumors and breast cancer (Álvarez et al., 2012). 5-FU has been used in clinical practice for decades and is currently considered the most cardiotoxic chemotherapeutic drug besides anthracyclines, as it can cause irreversible cardiac injury (Gradishar and Vokes, 1990). Its clinical manifestations include angina pectoris, palpitation, dyspnea, changes in blood pressure, and arrhythmia. The mechanisms of 5-FU-induced cardiotoxicity include induction of coronary artery spasm, damage to the vascular endothelium, and oxidative stress (Allison et al., 2020; Entezar-Almahdi et al., 2020; More et al., 2021). Studies have also shown that 5-FU can cause myocardial injury, fibrosis, and ventricular remodeling in animals (Tsibiribi et al., 2006; Li et al., 2021; 2022).

Platinum-based agents are a kind of effective broad-spectrum antitumor drug that exert their antitumor effects by interfering with DNA replication, inhibiting RNA and protein synthesis. Cisplatin is a first-generation platinum complex that is a first-line treatment for many solid tumors and one of the most widely used and strongest antitumor drugs. In addition, there are second-generation carboplatin and nedaplatin; and third-generation oxaliplatin and loplatin (Wachters et al., 2004; Oun et al., 2018). The cardiotoxicity of platinum-based agents has non-specific clinical manifestations, including arrhythmia, angina pectoris, acute myocardial infarction, cardiomyopathy, abnormal blood pressure, and thrombosis. Platinum-based agents can directly increase ROS in cardiomyocytes or induce oxidative stress and damage mitochondria, resulting in direct myocardial toxicity and impaired cardiac function (Wachters et al., 2004; Dilruba and Kalayda, 2016).

### 4.2 Nanotechnology-based formulations of other chemotherapy drugs in R&D

Currently, no nano formulations of fluorouracil have been approved for marketing, although several researchers have tried to various nano-delivery delivery systems with 5-FU to achieve better efficacy and reduce side effects. Solid lipid NPs (SLNs) loaded with 5-FU were prepared by Patel et al. through a temperature-regulated coagulation method. The anticancer activity of the 5-FU-loaded SLNs was investigated in the Caco-2 cell line, revealing a concentration-dependent decrease in cell viability. This study demonstrated that the SLN delivery system loaded with 5-FU had superior anticancer activity compared to conventional 5-FU (Patel et al., 2014). Other researchers developed chitosan-loaded 5-FU NPs to deliver a drug targeting the colon region in order to minimize its toxic effects on healthy cells. They concluded that the formulated chitosan NPs enhanced the ability of 5-FU to target the colon region and subsequently developed a sustained release over a prolonged period of 24 h, which effectively reduced the killing of healthy cells by the drug (Tummala et al., 2015). 5- FU citrus pectin NPs (CPNs) were fabricated and coated with Eudragit S100 (E-CPNs) for targeted delivery in colon cancer. In an *in vitro* cytotoxicity assay using HT-29 cancer cells, the CPNs exhibited a 1.5-fold greater cytotoxicity compared to a conventional 5-FU solution. *In vivo* data clearly showed that Eudragit S100 successfully protected the NPs, allowing them to reach the colon region. Subsequently, the NPs were absorbed by the colon and exhibited sustained release for a longer period of time (Subudhi et al., 2015). However, there are no direct or indirect experimental data in the above studies to support that nano-delivery systems can reduce 5-FU associated cardiotoxicity.

A liposomal formulation of Cisplatin, termed Lipoplatin, with an average diameter of 110 nm and a half-life of 120 h, has completed phase III clinical trials. This formulation, in combination with paclitaxel, showed much higher antitumor activity against NSCLC than cisplatin alone (Mochida et al., 2017). One study used PEGylated liposomes to co-deliver cisplatin and curcumin, which mitigated side effects and improved the anticancer activity of cisplatin against mouse hepatocellular carcinoma H22 and human HCC HepG2 xenograft models (Cheng et al., 2018). The co-delivery of irinotecan hydrochloride with oxaliplatin by a nanoliposome showed higher cytotoxicity compared with single-loaded liposomes of each drug toward CT-26 and HCT-116 cells as colorectal cancer cell lines. Comparatively, the antitumor activity after 22 days of treatment by the co-loaded liposomes was more than the synchronous and sequential administration of oxaliplatin and irinotecan (Zhang et al., 2016). Direct cardiotoxicity assessment experiments have not been designed in the study of platinum-based drug nano-delivery systems, so there are no experimental data directly confirming their cardiotoxicity-reducing effects.

## 4 Discussion

The development of antitumor nanomedicine requires huge investment, and there are many risks in the process of preclinical and clinical research. At present, few chemotherapeutic drug NPs have been successfully applied in clinical practice, and NPs based on cardiotoxic chemotherapeutic drugs are even fewer, as shown above (summarized in Table 1). Moreover, these drugs are non-molecularly targeted nano agents, which can only improve the distribution and elimination process of drugs *in vivo* through liposome encapsulation and albumin binding. However, these classic nanotechnologies are still effective at reducing cardiotoxicity.

**TABLE 1:** Summary of Nanotechnology-based marketed products of chemotherapeutic drugs (only best known chemotherapeutic agents that cause cardiotoxicity).

Trade name	Drug agent	Formulation type	Clinical applications	Company	Year of approval
Doxil	Doxorubicin	Pegylated liposome	Ovarian cancer, AIDS-related Kaposi’s Sarcoma.	Ben Venue Laboratories, TTY Biopharm.	1995
DaunoXom	Daunorubicin citrate	Liposome	Acute myeloid leukemia	Gilead Sciences	1996
Myocet	Doxorubicin	Non-pegylated liposome	Metastatic breast cancer	Enzon Pharmaceutical	2000
Lipo Dox	Doxorubicin	Pegylated liposome	AIDS-related Kaposi’s Sarcoma	Sun Pharma	2013
Abraxane	Paclitaxel	Albumin based NPs	Breast cancer	Abraxis BioScience	2005
Genexol-PM	Paclitaxel	Lyophilized polymeric micelles	Breast cancer, non-small cell lung cancer.	Samyang Corporation	2007
Marqibo	Vincristine sulfate	Liposome	Philadelphia chromosome-negative acute lymphoblastic leukemia	Talon Therapeutics	2012

Systemic delivery via the circulatory system is the most widely used method of drug delivery. Drugs can exit the circulation via cellular transport, paracellular transport, filtration in the kidney, the mononuclear phagocytic system (MPS), EPR effects, or defects in the vasculature (e.g., due to inflammation or injury). The particle size of existing NPs is too large to cross the endothelial system by transcellular transport. Paracellular transport at cell–cell junctions in the endothelium is limited to molecules that are ≤2 nm in size (Lundqvist et al., 2008; Dawidczyk et al., 2014). Tumor neo vasculature and inflamed or injured vascular endothelial are leaky and can lead to NPs exiting from circulation via the EPR effect. Consequently, the endothelial system of the blood vessels supplying blood to the heart is harder to penetrate compared to that at the tumor site, which may be one of the reasons why NP delivery systems are able to reduce the cardiotoxicity of encapsulated drugs. Conventional drugs typically reach peak plasma concentration (C_max_) during intravenous infusion, followed by a decrease in plasma drug concentration. Similarly, drug-encapsulated NPs reach Cmax during infusion, but the plasma concentration of the released drug is lower at this time and requires a period of slow release in the body circulation before reaching Cmax (Allen et al., 2006; Zhu et al., 2019). As NPs have a lower Cmax than conventional drugs, they can mitigate the toxicity induced by chemotherapy drugs associated with Cmax. Blood contains various proteins, molecules, and ions, along with red blood cells, leukocytes, and platelets. Human serum albumin, the most abundant protein in blood, is responsible for the transport of a wide range of molecules in the body. Antibodies, complement proteins, and circulating proteins such as mannose-binding lectin are examples of opsonins, a class of proteins that promote uptake by the MPS (Lundqvist et al., 2011). Modifying NPs with specific proteins enhances their delivery ability to specific organs and reduces their aggregation in healthy organs (Wang et al., 2019).

Although anthracyclic drugs have high antitumor effect, their cardiotoxicity is obvious, which seriously limits their application (Vejpongsa and Yeh, 2014; Alavi and Varma, 2020). Therefore, much research on the modification of anthracycline drugs via nanotechnology to reduce their cardiotoxicity has been performed, and many achievements have been made. Researchers have effectively increased the distribution ability of anthracycline drugs in target tissues and reduced their distribution in heart tissues with different nanodosage forms (Vejpongsa and Yeh, 2014; Skubitz et al., 2017). With the further development of nanotechnology, we predict that new anthracycline nanomedicines will achieve even lower cardiotoxicity.

As mentioned above, paclitaxel and vinblastine, while also known to be cardiotoxic, are less toxic than anthracyclines and are usually reversible (Lichtman et al., 2000; Gallego-Jara et al., 2020); therefore, although there are marketed nanomaterials based on them, the main indicators of investigation in the clinical trials do not include an evaluation of cardiac toxicity. In fact, this is a good direction to expand the content of clinical trials, whether in real-world studies of marketed varieties or phase II/III clinical studies of unmarketed varieties. This is especially true in clinical trials of multidrug regimens, in which the risk of heart damage is higher. We expect that more treatment regiments in this class of nanomedicines will be proven to reduce cardiotoxicity and benefit more patients.

For the other chemotherapy drugs with cardiotoxicity, such as 5-FU and cisplatin, there is still a long way to go before nanotechnology can reduce their cardiotoxicity clinically. First, as mentioned above, nanomedicines are expensive to develop. Second, even if the drugs are modified via nanotechnology, their overall performance may not be better than those of the new generation similar drugs developed by chemical means. For example, from 5-FU evolved into capecitabine (Saif et al., 2008) and from cisplatin evolved into oxaliplatin, lobaplatin (Dilruba and Kalayda, 2016). Future research is needed to answer these questions.

In general, nanomedicine is an effective method to reduce the cardiotoxicity of traditional chemotherapy drugs. However, cardiovascular complications in cancer treatment are very complex diseases, requiring the application of multiple measures, including screening, application of protective drugs, modification of chemotherapy drugs, and improvement of combination medication regimen, so as to achieve effective management and prevention.
